# Optimizing optical, dielectric, and electrical properties of polyvinyl alcohol/polyvinyl pyrrolidone/poly(3,4-ethylene dioxythiophene) polystyrene sulfonate/NiO-based polymeric nanocomposites for optoelectronic applications

**DOI:** 10.1038/s41598-024-76918-5

**Published:** 2025-01-04

**Authors:** E. Salim, A. Magdy, A. H. EL-Farrash, A. El-Shaer

**Affiliations:** 1 Physics Department, Faculty of Science, Mansoura University, Mansoura, 35516 Egypt; 2 Physics Department, Faculty of Science, Kafrelsheikh University, Kafrelsheikh, 33516 Egypt

**Keywords:** Nanocomposite, PEDOT:PSS, Bulk resistance, Dielectric constant, NiO, Materials science, Optics and photonics

## Abstract

An electro- and optically favorable quaternary nanocomposite film was produced by solution-casting nickel oxide nanoparticles (NiO NPs) into polyvinyl alcohol (PVA), polyvinyl pyrrolidone (PVP), and poly(3,4-ethylenedioxythiophene) polystyrene sulfonate (PEDOT/PSS). Based on transmission electron microscopy (TEM) and X-ray diffraction (XRD) observations, the synthesized NiO NPs have a cubic phase and a diameter between 10 and 45 nm. The complexity and interactions observed through XRD patterns, UV–visible spectra, and FTIR measurements suggest that the NPs are not just dispersed within the polymer matrix, but are interacting with it, leading to enhanced dielectric properties and AC electrical conductivity. From 9 × 10^3^ to 3.22 × 10^3^ Ω, NiO NPs concentrations reduce bulk resistance Rb, indicating more linked conductive channels. The dielectric tests showed that polarized nanoparticles increased polarizability under electric field conditions. The incorporation of NiO NPs boosted DC conductivity from 1.25 × 10^–6^ to 5.64 × 10^–5^ S m^−1^. The mobility of NiO NPs boosts DC conductivity linearly with field frequency. These interactions can lead to improved electrical conductivity, energy storage capabilities, and overall efficiency of the nanocomposite, making it a promising material for various applications.

## Introduction

Currently, nanocomposite materials are attracting a lot of attention because of their unique properties. By combining two or more materials, the properties of each component are enhanced, creating materials that are stronger and more durable than either of the individual components. Furthermore, these nanocomposites can be tailored to have specific properties that make them useful for a variety of applications, including light-emitting devices^1,2^, sensors^3^, and direct methanol fuel cells^4^. There are several important synthesis methods for nanocomposite formation, including electrospinning^5^, sol–gel^6^, in situ processes^7^, casting techniques^8^, and pyrolysis^9^.

PVA is a semicrystalline polar polymer with hydroxyl groups that may form hydrogen bonds with different fillers in polymeric systems. PVA possesses strong dielectric properties, a high capacity for charge storage, and optical and dielectric features. So, PVA can be used for different applications such as optoelectronics and electronics sectors^10–13^. Furthermore, PVP is an amorphous, water-soluble, and non-toxic polymer. PVP has also drawn the attention of researchers because of its special qualities, which include its inexpensive cost, good dielectric nature, water solubility, and mechanical strength^14,15^. The presence of N-O, and C-O groups permits it to generate various complexes through covalent interactions with inorganic salts and nanofillers on their surface, which provide their stabilization without accumulations^16^. To improve the conductivity of the polymer blend, a conductive polymer may be incorporated. This additive not only enhances the dispersibility of the conductive particles but also increases the overall conductivity of the blend, making it suitable for various applications in electronics and energy storage^17–19^. PEDOT/PSS has superior electrochemical stability, high electrical conductivity (σ), and film-forming properties among these polymers^20^. The improvement of both the miscible polymer blend and its electrical conductivity results from the function groups involved in the backbone’s polymer chain^21^. In addition, the incorporation of nanoparticles within the polymer blend creates molecular bridges that enable the formation of nanocomposites. These nanoparticles’ uniform dispersion and distribution typically lead to the creation of large polymeric/filler interfacial areas in the composite, which is beneficial for the material’s electrical and dielectric properties^22,23^. Recently, scientists have gained a great interest in transition metal oxides because of their low cost, stability, and optoelectronic properties^24–26^. Reddy et al.^27^ prepared PVA/PEDOT: PSS/Copper (II) Oxide (CuO) nanocomposites with 15% CuO NPs, revealing good interaction and enhanced dielectric properties, suggesting potential energy storage applications due to their enhanced properties. The study also found that the higher dielectric constant, lower dielectric loss, and enhanced AC conductivity (*σ*_ac_ = 1.67 × 10^–5^ S m^−1^ at 150 °C) decrease impedance and capacitive reactance with increased nanofiller content. NiO NPs, one of the most significant metal oxides, are characterized by their thermal stability and photostability, high melting (~ 1955 ◦C), refractive index of ≈ 2.2, and a wide optical bandgap (E_g_ = 3.4-4 eV)^28,29^. As a result, NiO NPs are suitable for a variety of applications, including gas sensors, batteries, electrochemical capacitors, magnetic materials, and catalysis^30^. NiO NPs can be incorporated into the thin film layer of the solar cell, enhancing its light absorption and improving the overall efficiency of the device^30,31^.

In this work, flexible quaternary polymeric nanocomposite films were created by solution-casting PVA, PVP, PEDOT/PSS, and NiO NPs. An extensive investigation was reported to compare the effects of various concentrations of NiO NPs on the electrical and dielectric properties of quaternary nanocomposite films.

## Experimental section

### Materials

PVA (Mw 31,000–50,000 g/mol), PVP (Mw 40,000 g/mol), and PEDOT/PSS (1.3 wt % dispersion in H_2_O) were all bought from Sigma-Aldrich. Nickel chloride hexahydrate (Nicl_2._ 6H_2_O) and sodium hydroxide (NaOH) were obtained from Merck.

### NiO NPs synthesis

The precipitation procedure was used to produce NiO NPs. In the first stage, a magnet stirrer was used to thoroughly dissolve nickel chloride hexahydrate and sodium hydroxide in deionized water. A molar ratio of 1:2 between the two aqueous solutions was gradually added drop by drop at room temperature T = 303 K under vigorous stirring. In the next stage, the precipitate was collected, filtered, and washed several times with deionized water. As a result of the precipitation process, the precipitate was collected, filtered, and rinsed with deionized water. In an oven at 363–368 K, it was dried for two days before being ground into a fine powder by agate mortars. After obtaining a fine powder, it was heated for four hours at 723 K, then ground for a second time. The particle size, on average, is 18.2 nm.

### NiO/PVA/PVP/PEDOT/PSS nanocomposite fabrication

Separately, 1 g of PVP and 1 g of PVA were completely dissolved in 100 mL deionized water at room temperature and 50 °C, respectively, and then mixed and stirred (300 rpm) for 6 h. Then, five equal volumes of 20 ml of dissolved PVP/PVA and 0.5 mL of PEDOT/PSS were added and stirred for 1 h. After that, 0.0, 0.25, 0.5, 0.75, and 1.0 wt % of NiO NPs were dropped into the mixtures and stirred for 2 h. In the final step, the mixtures were cast into 55 mm diameter plastic Petri dishes and dried at 50 °C. The thickness of the films ranges from about 240 nm.

### Characterization

Nanocomposite films were investigated using Nicolet iS10 FTIR spectroscopy in the 400 – 4000 cm^−1^ wavenumber range. XRD patterns were examined with a DIANO X-Ray Diffractometer using CuKα radiations. A UV-visible spectrophotometer was used to measure the optical absorption of the films. Japan’s JEOL/JEM/1011 TEM was used to examine NiO nanoparticles’ size and form. At 303 K, broadband dielectric spectroscopy (Novo Control Turnkey Concept 40 System) was used to measure the dielectric properties and impedances of the films. The EIS spectrum analyzer 1.0 is used to fit the impedance curves.

## Results and discussion

### TEM of NiO NPs

**Fig. 1:**
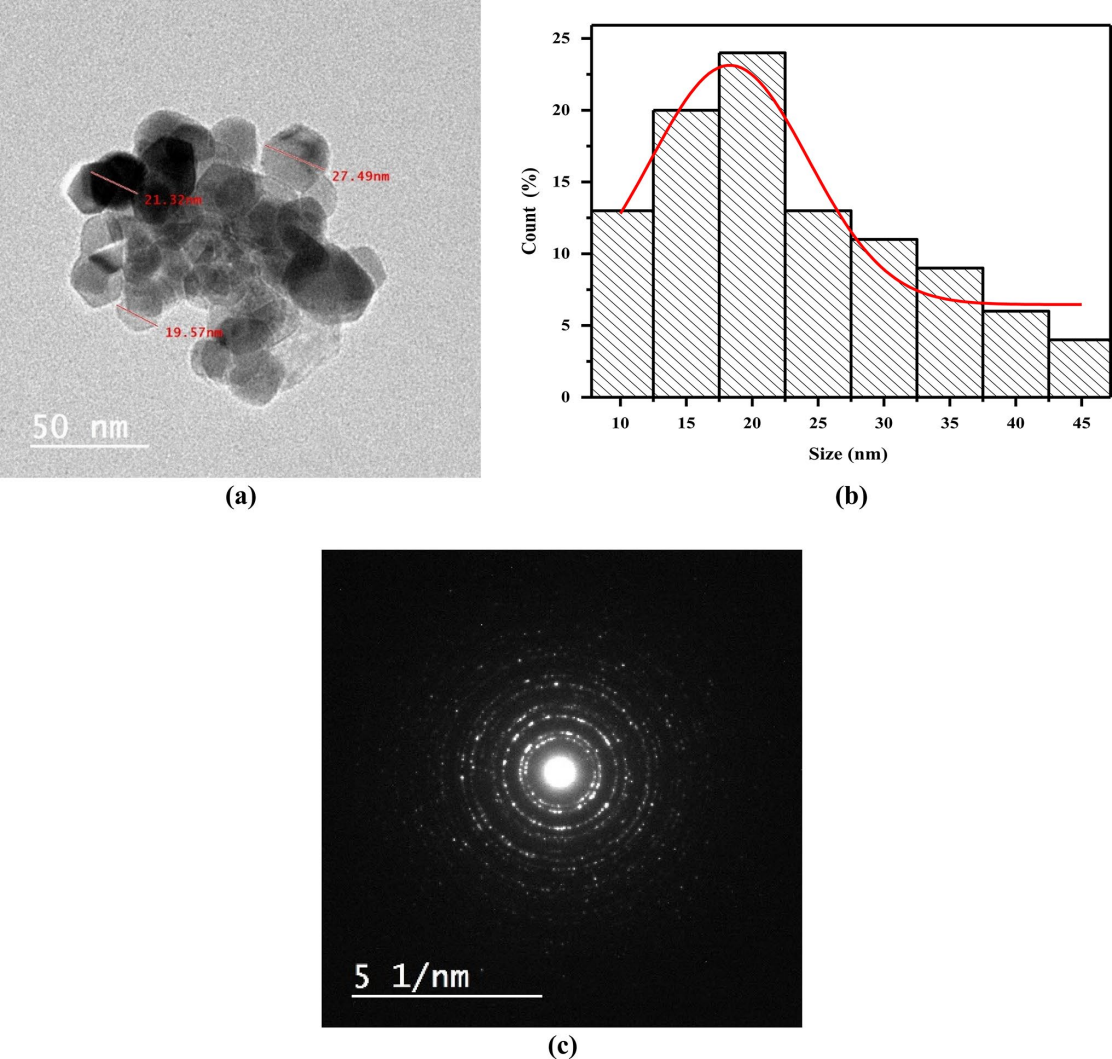
(**a**) TEM image, (**b**) histogram distribution plot, and, (**c**) diffraction pattern of NiO NPs.

Figure 1a illustrates the TEM image of NiO NPs with a cubic (hexagonal) structure of about 18.2 nm, based on the histogram distribution plot (Fig. 1b). The strong diffraction pattern of NiO NPs in Fig. 1c confirms the high degree of crystallinity of NiO NPs.

### X-ray diffraction analysis

**Fig. 2:**
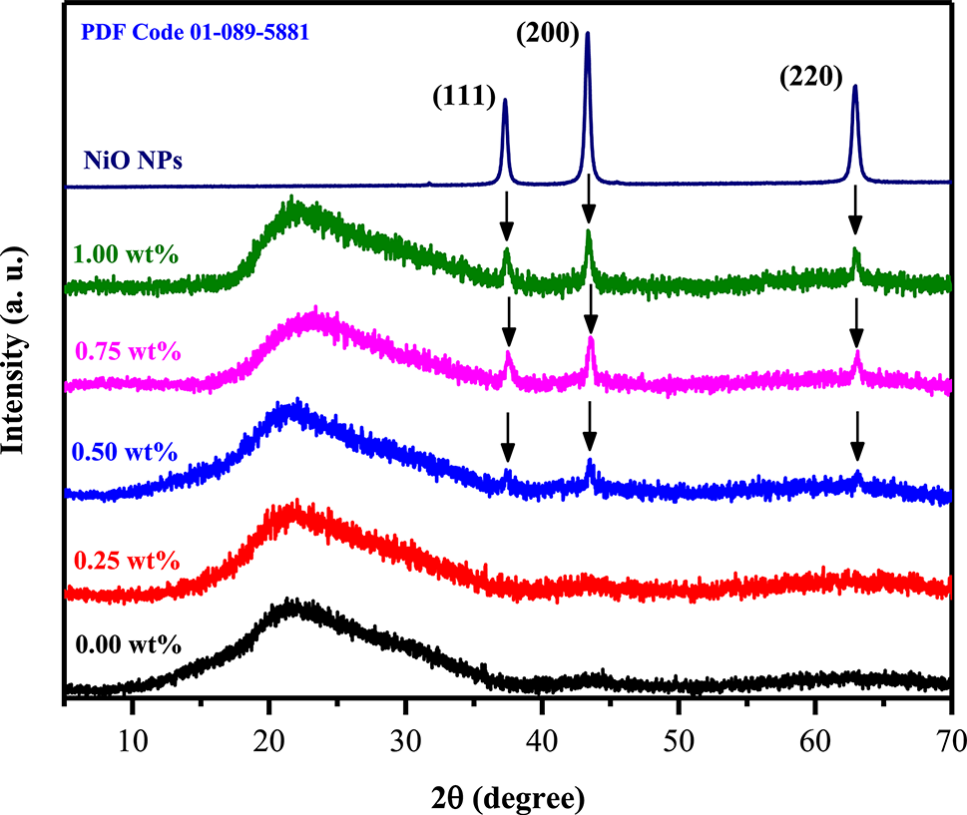
XRD analysis of pure NiO NPs as well as PVA/PVP/PEDOT/PSS with various NiO contents.

XRD measurements are used to examine the crystal structure of nanocomposite samples. Figure 2 depicts the XRD pattern of NiO NPs, pure PVA/PVP/PEDOT/PSS, and various content NiO-filled PVA/PVP/PEDOT/PSS. A single broad diffraction peak was observed at 2θ = 22.26°, indicating high compatibility between the blend components. The diffraction peaks for pure NiO NPs were observed at 2θ = 37.31°, 43.42°, and 63.82°, which correspond respectively to the (111), the (200), and the (220) planes^32^. The observed peaks confirm that NiO NPs with high purity form a cubic structure. The crystal size (D) of prepared NiO NPs was calculated using Scherrer’s equation ${\text{D}} = \frac{{0.9{\uplambda }}}{{{\upbeta }\cos {\uptheta }}}$ where ${\uplambda }$ is the x-ray wavelength, ${\upbeta }$ is FWHM and ${\uptheta }$ is the diffraction angle^33^ listed in Table 1. In accordance with TEM results, it was found that the particle size is approximately 21 nm. As a result of NiO NP doping, the intensity and position of the abroad diffraction peak in the doped samples have changed slightly. The crystallinity (X_c_) of the nanocomposite films is determined using the following formula^34^:

1 $${\text{X}}_{{\text{c}}} \left( \% \right) = \frac{{A_{peak} }}{{A_{peak} + A_{hump} }} \times {1}00$$

where A_peak_ and A_hump_ represent the areas of crystalline and hump peaks. According to the X_c_ values, pure and NiO-filled nanocomposite films showed 56.15, 52.64, 45.75, 41.37, and 39.85%. As a result of the considerable variation in the polymer-nanofiller interaction, the decrease in X_c_ value indicates the formation of more amorphous regions within the nanocomposite films. This increase in amorphousness may explain the enhanced ionic diffusivity that is associated with high ionic conductivity^35^. Highly doped nanocomposite samples demonstrated three diffraction peaks of NiO NPs, confirming the presence of NiO NPs in these samples. As a consequence, the addition of NiO NPs to the PVA/PVP/PEDOT/PSS matrix supports the FTIR results, as detailed below.

**Table 1:** The NiO NPs lattice parameters.

2θ (^o^)	h k l	d-spacing (Å)	FWHM	D (nm)
37.25	111	2.4114	0.4394	19.92
43.29	200	2.0885	0.4706	18.96
62.86	220	1.4771	0.5760	16.87

### FTIR

**Fig. 3:**
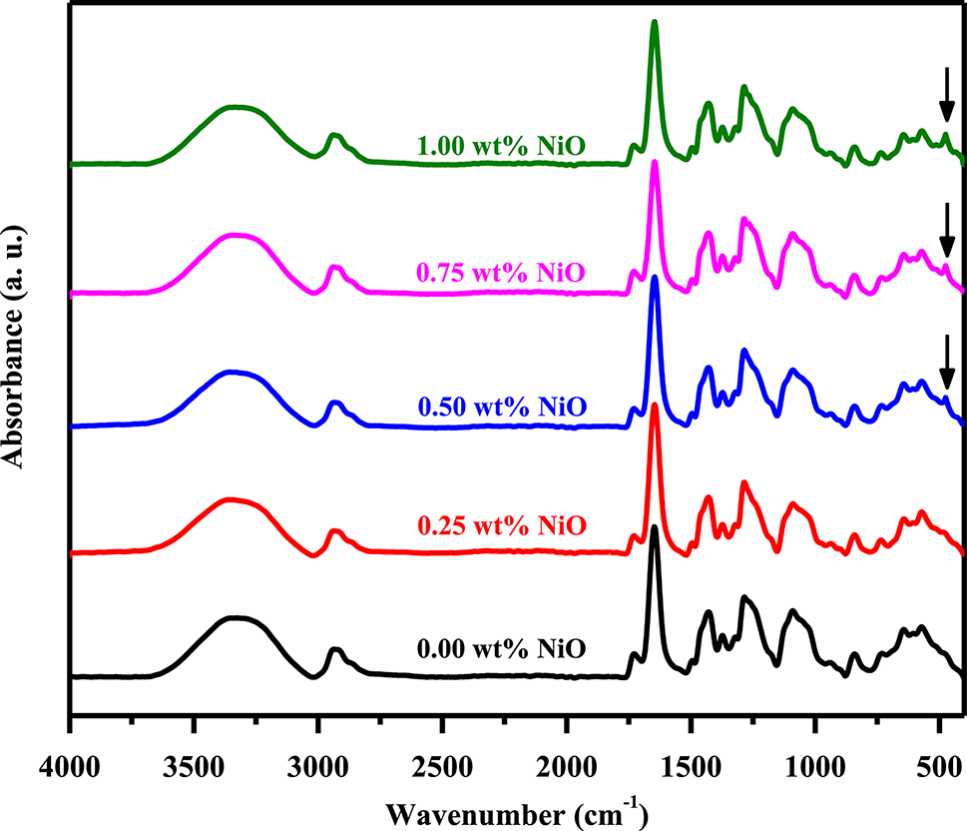
FTIR analysis of pure PVA/PVP/PEDOT/PSS and NiO/PVA/PVP/PEDOT/PSS with varying NiO content.

FTIR spectra of pure and various percentages of NiO filled with PVA/PVP/PEDOT/PSS are shown in Fig. 3. The spectra of prepared films showed absorption bands associated with the bending and stretching vibrations generated by functional groups. A summary of these bands are listed in Table 2. There are vibration bands at 1386 cm^−1^, 1274 cm^−1^, and 843 cm^−1^ that correspond to the vibrations of CH_2_ bending^36,37^. The vibration bands at 2943 cm^−1^ correspond to the vibrations of CH_2_ symmetric stretching^38^ and 1088 cm^−1^ correspond to the vibrations of CO stretching of the characteristic vibration bands of PVA^38^. The peak at 1438 cm^−1^ is attributed to the C–N stretching vibration absorption of PVP^39^. PEDOT/PSS exhibited a band of absorption at 1647 cm^−1^ due to C=C stretching and 3322 cm^−1^ due to OH stretching^39,40^. The peak at 568 cm^−1^ can be attributed to N-C=O bending from PVP^39,41^. Upon adding NiO NPs with different concentrations to PVA/PVP/PEDOT/PSS, a small peak occurred at 475 cm^−1^ due to Ni-O vibrations. This peak increases slightly with increasing NiO concentration. These significant changes in peak intensities and extensive variations in a few peaks indicate polymer-nanoparticle interactions in nanocomposite films.

**Table 2:** FTIR vibration modes of NiO/PVA/PVP/PEDOT/PSS nanocomposites.

Wavenumber (cm^−1^)	Vibration mode	Source	Reference
3322	O-H stretching	PEDOT/PSS, PVP, PVA	^39,40^
2943	CH_2_ symmetric stretching	PVA	^38^
1737	C=O stretching	PVA	^38^
1647	RC=C stretching	PEDOT/PSS	^40^
1438	C–N stretching vibration	PVP	^39^
1386	CH_2_ bending vibrations	PVA	^36^
1274	CH_2_ bending vibrations	PVA	^37^
1088	CO stretching	PVA	^37^
843	CH_2_ bending vibrations	PVA	^36^
649	C-N bending	PVP	^42^
568	N-C=O bending	PVP	^39,41^
425	Ni-O vibration	NiO	^42^

### UV-VIS Absorption

**Fig. 4:**
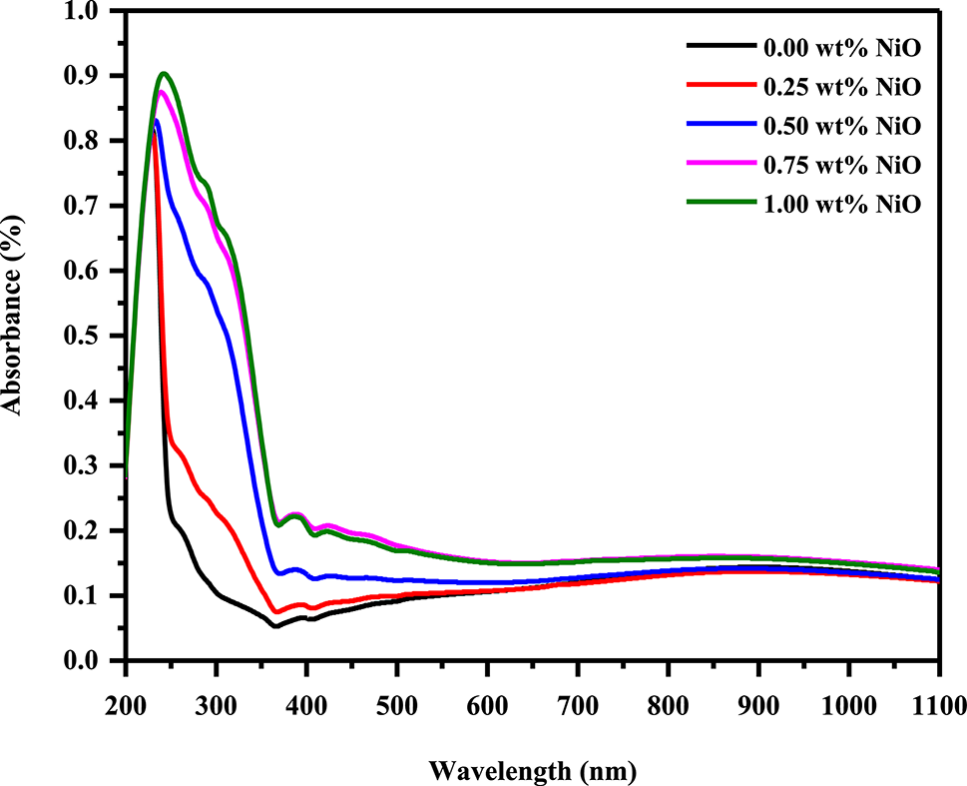
UV-Vis absorbance of pure PVA/PVP/PEDOT/PSS and filled with different NiO content.

Figure 4 shows the absorption spectra of pure PVA/PVP/PEDOT/PSS and NiO-filled PVA/PVP/PEDOT/PSS nanocomposite with different NiO contents between 200 and 1100 nm. The spectrum of pure film displayed an absorption peak at 227.5 nm attributed to the π-π^*^ transition^43^ due to C=C of pure PVA/PVP/PEDOT/PSS^44^. Upon the addition of NiO Nps, the absorbance of the NiO-filled PVA/PVP/PEDOT/PSS nanocomposite samples increased, and its position shifted to 244 nm which can be explained by a change in band gap energy and is correlated to the modification in the crystallinity of the films. Moreover, there are another two small absorption peaks at 391 and 425 nm, which may be attributed to NiO nanoparticles^45^.

**Fig. 5:**
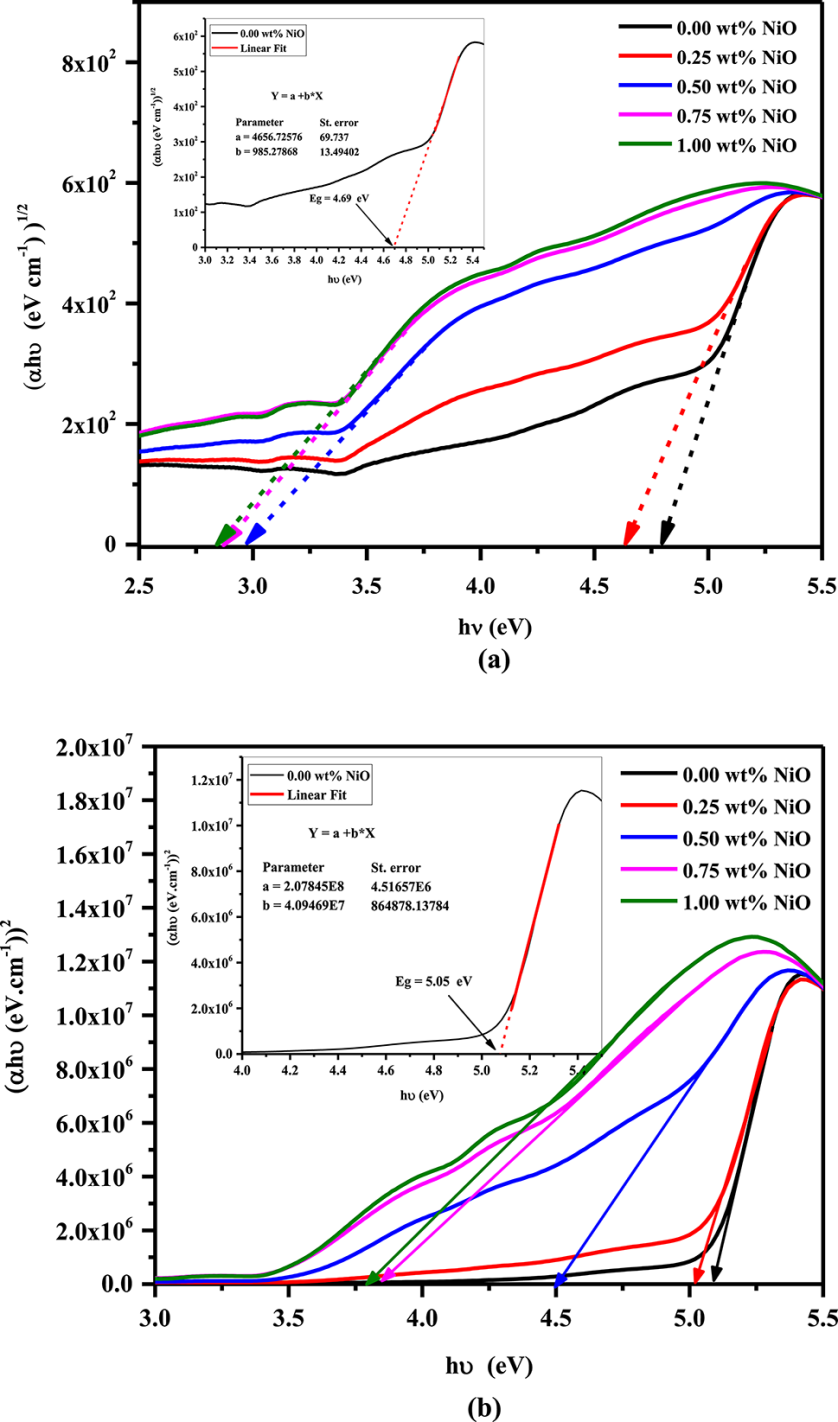
(**a**) (αhυ)^1/2^ vs hυ (inset plot of band gap energy calculation via linear fitting) and (**b**) (αhυ)^2^ vs hυ (inset plot of band gap energy calculation via linear fitting) of NiO/PVA/PVP/PEDOT/PSS with varying NiO content.

The nature of optical transitions in the nanocomposite films is examined by the Tauc equation as follows^46^:

2 $$\upalpha {\text{h}}\upupsilon = {\text{B}}({\text{h}}\upupsilon - {\text{E}}_{{{\text{g}} }} )^{{\text{m}}}$$

where B is a constant, *α* is the absorption coefficient (α = 2.303 $\frac{{\text{Absorbance}}}{{{\text{Thickness}}}}$), and m is an exponent that characterizes the nature of the electronic transition nature, i.e., for the direct allowed transition (m = 1/2), and indirect allowed transition (m = 2). Figure 5a and b depicts the dependence of (αhυ)^1/2^ and (αhυ)^2^ on hυ for all samples. Table 3 shows the calculated values of direct (${\text{E}}_{{\text{g}}}^{{\text{d}}}$) and indirect (${\text{E}}_{{\text{g}}}^{{\text{i}}}$) band gap energies via linearly fitted and intercept on the hυ axis. It is observed that the indirect and direct energy gaps decrease with increasing NiO NP concentrations. The changes in the energy gap values could be attributed to the presence of NiO NPs, which introduced polaronic and defect levels that altered the electronic structure of the polymeric matrix^47^. Consequently, the quantity of these defects and the concentration of NiO NPs are related to the density of localized states N(E). There is evidence that the spread of localized states with various colored centers in the mobility gap may be triggered by an increase in NiO NPs concentrations^30,31^. Additionally, this increase in localized states may indicate that the filled samples have a lower degree of crystallinity^48^. Based on XRD and electrical conductivity measurements, these results are consistent with those obtained. The enhanced optical energy values suggest electrochemical and optoelectronic applications.

**Table 3:** The band gap energy and linear refractive index values of NiO/PVA/PVP/PEDOT/PSS nanocomposite.

NiO NPs @ Sample (wt%)	${\mathbf{E}}_{{\mathbf{g}}}^{{\mathbf{i}}}$(eV)	${\mathbf{E}}_{{\mathbf{g}}}^{{\mathbf{d}}}$(eV)	${\mathbf{n}}_{{\mathbf{o}}}^{{\mathbf{i}}}$	${\mathbf{n}}_{{\mathbf{o}}}^{{\mathbf{d}}}$
0.00	4.69 $\pm 0.14$	5.08 $\pm 0.05$	2.03	1.98
0.25	4.50 $\pm$ 0.13	5.01 $\pm$ 0.07	2.05	1.99
0.50	2.91 $\pm 0.02$	4.49 $\pm$ 0.04	2.40	2.06
0.75	2.81 $\pm$ 0.04	3.84 $\pm$ 0.03	2.43	2.18
1.00	2.79 $\pm$ 0.06	3.79 $\pm$ 0.02	2.44	2.19

Among the most essential optical properties, the refractive index n plays a significant role in practical applications. It governs a wide range of optoelectronic and electronic applications, including LEDs, waveguides, photodetectors, and filters. Based on the optical band gaps, the Dimitrov-Sakka equation can be used to calculate the linear refractive index n_o_^49^:

3 $$\frac{{{\text{n}}_{{\text{o}}}^{2} - 1}}{{{\text{n}}_{{\text{o}}}^{2} + 2}} = 1 - \sqrt {\frac{{{\text{E}}_{{\text{g}}} }}{20}}$$

As indicated in Table 3, an increase in NiO NPs results in a rise in refractive index values. The increased refractive index of nanocomposite films after incorporating NiO NPs could be the result of structural changes in the polymeric matrix.

### Conductivity analysis

The mobility of charge carriers is an important component in influencing the electrical conductivity of amorphous polymers. Tunable capacitors, sensors, and RF filters can all benefit from frequency dependency. Furthermore, the approach may be applied to enhance the electrical characteristics of complicated nanocomposites. By applying AC voltages across the sample at various frequencies, impedance spectroscopy can be used to determine the AC conductivity of samples:

4 $$\sigma_{ac} = 2f\varepsilon^{\prime}\varepsilon_{o} tan$$

**Fig. 6:**
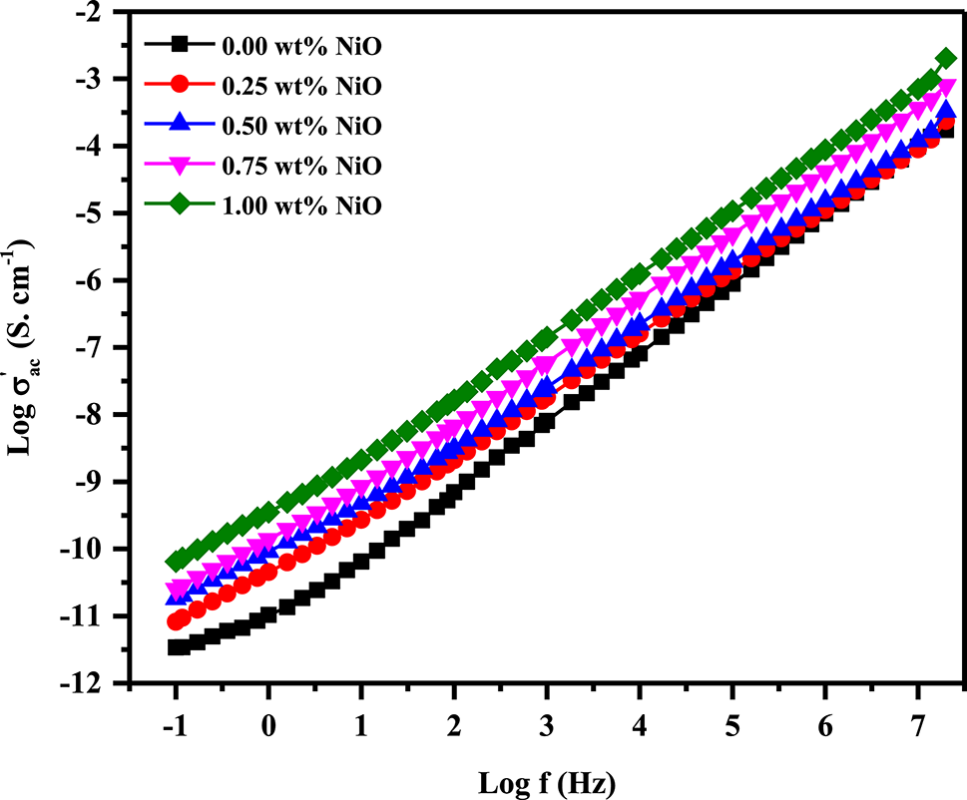
The changes in Log (σ_ac_) versus Log f for pure and NiO-filled nanocomposite films.

Specifically, tanδ refers to the dissipation factor, f refers to the frequency of the applied signal, and ε′ and ε_o_ represent the dielectric constants of the material and free space, respectively. Figure 6 shows the frequency response of PVA/PVP/PEDOT/PSS loaded with different percentages of NiO NPs at ambient temperatures. Based on the figure, it appears that conductivity increases with NiO NPs content since more current paths are provided by the dispersed NiO NPs. Such nanocomposites exhibit varying conductivities depending on the shape, type, size, and dispersion of nanofillers^50,51^. Amorphous polymers allow more contact points between nanoparticles and polymer matrix, increasing electron and ion pathways. This improves the conductivity of nanocomposite by allowing electrons and ions to travel easily through it^45^.

A low-frequency system has a nearly constant conductivity, but a high-frequency system follows the power-law equation of Jonscher^52^:

5 $$s_{ac} \left( \omega \right) = s_{dc} + A\omega^{s}$$

In this scenario, *σ*_*dc*_ represents DC conductivity (at $\omega$ ≈ 0), A represents a frequency-dependent component that affects the degree of polarizability, and s represents a temperature and frequency-dependent exponent.

According to Jonscher, a 'universal dynamic reaction’ may be applied to a wide range of materials^45^. Low-frequency ranges are associated with bulk conductivity, which is generated by charge carrier displacements and hence connected to DC conductivity^51^. Charge carriers’ conductivity improves linearly with frequency because higher frequencies allow them to travel more easily than lower frequencies^53^. At higher frequencies, the loss factor dominates, resulting in a relative increase in conductivity^54^. The *σ*_*dc*_ value exhibited an increase from 1.25 × 10^–6^ to 5.64 × 10^–5^ S.m^−1^ with the addition of NiO NPs.

### Dielectric characteristics

The dielectric constant (ε′) of a material is a measurement of its electrical properties, and it is a measure of how strongly the material can store an electrical charge. Material with a higher dielectric constant can store more electrical energy. The dielectric loss (ε″) is the amount of energy lost as heat or radiation when a substance is exposed to an electric field. It is an important measure for materials that are used in electrical applications, as it determines the efficiency of the material. A low dielectric constant provides excellent insulation for high-frequency applications, reducing the amount of energy lost as heat^55^. Following are the equations used to calculate dielectric constants and loss values:

6 $$\upvarepsilon \prime = \frac{Cd}{{\upvarepsilon _{0} A}}$$

7 $$\varepsilon \prime \prime = \frac{\sigma }{{\upomega \upvarepsilon _{0} }}$$

**Fig. 7:**
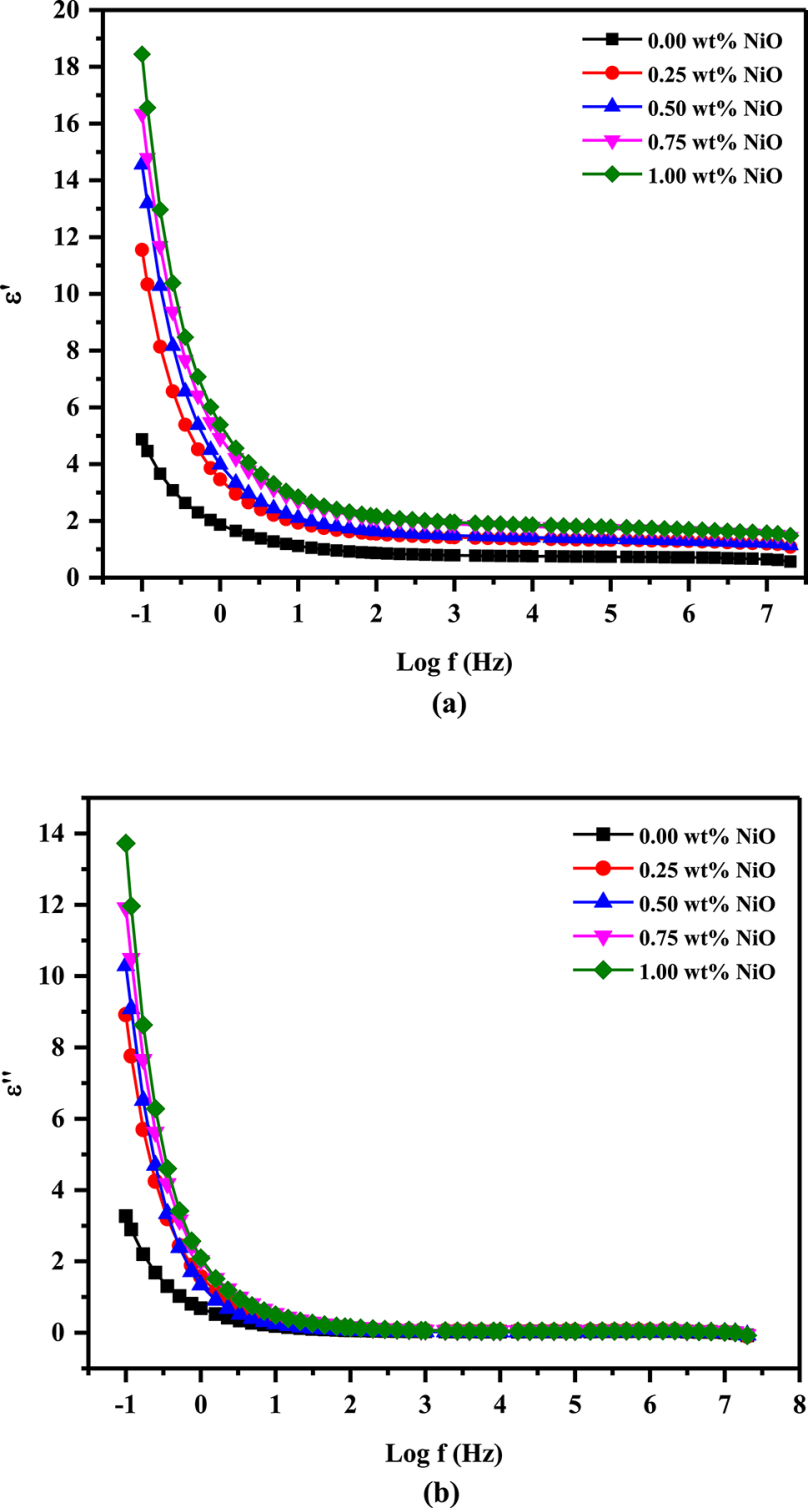
Frequency dependence of the (**a**) real and (**b**) imaginary part of the dielectric constant of the nanocomposite films.

Here, d, A, and C represent sample thickness, cross-sectional area, and capacitance, respectively. PVA/PVP/PEDOT/PSS nanocomposites filled with NiO NPs show changes in ε′ and ε″ over frequency as illustrated in Fig. 7a and b. As a result, higher frequencies have a reduced effect on ε′ and ε″ values. At low frequencies, the high ε′ and ε″ values may be attributable to electrode and interface impacts, which account for the bulk of samples. Polar materials have high ε′ and ε″ values at low field frequencies but decline as field frequencies grow due to dipoles’ inability to keep up with variations^35,40^. Upon filling the NiO NPs, ε′ and ε″ values increased across all frequency ranges. With increasing NiO NPs concentrations, more of these nanocapacitors are produced, resulting in greater dielectric constants. Nano-fillers also increase the dielectric constant of dipoles by lowering limitations on their response to electrical fields^56^. On the other hand, NiO NPs and the dielectric matrix may polarize due to Maxwell–Wagner-Sillars (MWS) phenomena. A mismatch in conductivity or permittivity between the NPs and the matrix causes this polarization, which improves dielectric characteristics at lower frequencies^57^.

### Complex electric impedance

The complex impedance analysis of the samples under investigation can be obtained using the equation below^58^:

8 $$Z^{*} = Z^{\prime} - iZ^{\prime\prime} = \frac{1}{{i\omega \varepsilon_{o} \varepsilon^{*} }}$$

**Fig. 8:**
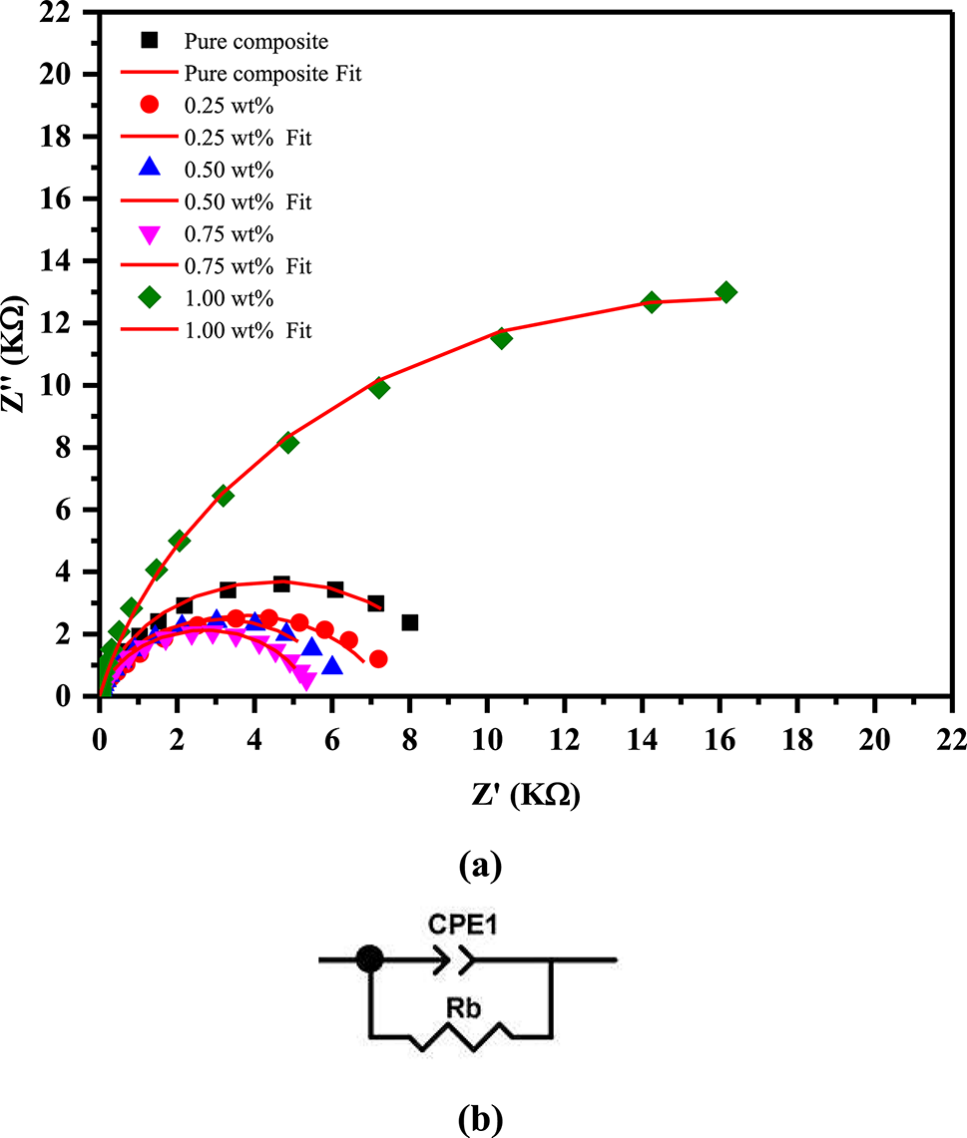
(**a**) The examined samples’ Nyquist plots and (**b**) their equivalent circuit.

The real and imaginary components of the complex impedance are denoted by Z′ and Z′′, respectively. Figure 8a shows Nyquist plots for the films, which were measured at room temperature and covered a broad range of frequencies between 10^–1^ Hz and 10^7^ Hz. Nyquist curves generate semicircular arcs with their centers located below Z’. In this semicircular arc, stationary polymer chains produce bulk capacitance, while ions’ motion produces bulk resistance^59^. The semicircular arc’s depression in its center indicates that the ionic relaxation mechanism is not Debye. A departure from the Debye type can be caused by uneven thicknesses and morphologies of the investigated films, as well as rough electrode surfaces. Nanocomposites’ Debye-type behavior may be affected by film thicknesses and morphologies. Different film thicknesses alter charge buildup and dispersion, influencing dielectric response. Additionally, rough electrode surfaces might complicate charge transport pathways, resulting in non-ideal dielectric behavior^60^.

The diameter of the semicircle decreases as the concentration of NiO NPs rises, demonstrating that bulk resistivity decreases while ionic conductivity increases.

Figure 8b shows an equivalent circuit with resistors and capacitances to demonstrate the impedance link between microstructure and electrical properties. Bulk Resistance (Rb) and Partial Capacitance (CPE) are used in parallel to form this circuit. This CPE’s impedance can be estimated as follows^61^:

9 $$Z_{CPE} = 1/Q(i)^{n}$$

CPE capacitors have diverged from purity as shown by this relationship, where Q represents 1/|Z| at 1rad/s, and n represents the element phase. At n = 1, the CPE functions as a pure capacitor, and at n = 0, it acts as a pure resistor.

According to Fig. 8a, the parameters of the equivalent circuit are listed in Table 4. The bulk resistance Rb lowers with increasing concentrations of NiO NPs, suggesting more interconnected conductive channels^62^. Additionally, bulk capacitance values (Q) rise with increasing concentrations of NiO NPs. The equivalent circuit selected is reasonably compatible with the data obtained.

**Table 4:** The fitting of equivalent circuit parameters.

NiO NPs @ Films (wt%)	Fitting parameters		
	Rb (ohm)	Q (F)	N
0.00	9.00 × 10^3^	3.08 × 10^–5^	0.88
0.25	7.43 × 10^3^	4.53 × 10^–5^	0.78
0.50	6.22 × 10^3^	4.64 × 10^–5^	0.86
0.75	5.53 × 10^3^	5.35 × 10^–5^	0.84
1.00	3.22 × 10^3^	7.96 × 10^–5^	0.85

## Conclusion

Nanocomposite films with different synthesized NiO NP contents were created by solution-casting PVA/PVP/PEDOT/PSS blends. According to XRD and TEM analyses, the NiO nanoparticles have a cubic structure of approximately 18.2 nm. Incorporating NiO NPs into PVA/PVP/PEDOT/PSS reduces the nanocomposites’ crystallinity. A distinctive diffraction peak confirmed the presence of NiO NPs. FT-IR spectra indicate the complexation between NiO NPs and polymeric composite. The bandgap energies of the nanocomposite films dropped with an increase in NiO NPs content, from 4.69 $\pm$ 0.14 eV to 2.79 $\pm$ 0.06 eV for indirect bandgaps and 5.08 $\pm$ 0.05 eV to 3.79 $\pm$ 0.02 eV for direct bandgaps. Impedance spectroscopy demonstrated that higher NiO NP concentrations increased the nanocomposite’s dielectric properties and AC conductivity. The equivalent electrical circuits’ impedance components Z′ and Z′′ were examined. Therefore, the decrease in the bulk resistance implies a stronger electrical pathway inside the polymeric materials. The nanocomposite films exhibited different electrical and optical properties depending on the NiO NPs content.

## Data Availability

The corresponding author is responsible for providing reasonable access to the datasets used in this study.
